# Rare Complications of Lumboperitoneal Shunts: Abdominal Cerebrospinal Fluid Pseudocysts in Adults

**DOI:** 10.7759/cureus.78225

**Published:** 2025-01-30

**Authors:** Tatsuya Tanaka, Tomoyuki Naito, Takahiro Kumono, Eiichi Suehiro, Takashi Agari, Kimihiro Nakahara, Kazuaki Shimoji, Hiroshi Itokawa, Keisuke Onoda, Akira Matsuno

**Affiliations:** 1 Department of Neurosurgery, International University of Health and Welfare Narita Hospital, Narita, JPN; 2 Department of Neurological Surgery, International University of Health and Welfare, Shizuoka, JPN

**Keywords:** abdominal pseudocyst, adult, cerebrospinal fluid, complication, lumboperitoneal shunt, ventriculoatrial shunt, ventriculoperitoneal shunt

## Abstract

Abdominal cerebrospinal fluid (CSF) pseudocysts are rare complications of shunt surgery, predominantly reported in pediatric patients undergoing a ventriculoperitoneal (VP) shunt. In contrast, their occurrence following lumboperitoneal (LP) shunt is exceptionally uncommon. We report the case of a 76-year-old woman who presented with recurrent symptoms, including gait disturbance and cognitive decline, approximately three years after undergoing LP shunt placement for idiopathic normal pressure hydrocephalus. Diagnostic imaging, including shuntography and abdominal computed tomography (CT), revealed an abdominal CSF pseudocyst, likely due to peritoneal adhesions from prior abdominal surgery. Reoperation with catheter repositioning successfully relieved her symptoms. This case highlights the necessity of considering abdominal CSF pseudocysts as a differential diagnosis for shunt malfunction, particularly in patients with a history of abdominal surgery. Early diagnostic procedures, such as shuntography and abdominal CT, are critical for timely intervention and symptom resolution.

## Introduction

A cerebrospinal fluid (CSF) shunt is a widely used treatment for hydrocephalus. However, several complications associated with this procedure have been reported. Abdominal CSF pseudocysts are rare complications characterized by fluid-filled sacs that result from CSF encapsulation in the peritoneal cavity. These pseudocysts can lead to shunt dysfunction, abdominal symptoms, and increased intracranial pressure [1,2]. The incidence of abdominal CSF pseudocysts is reported to be 1%-4.5% in patients with ventriculoperitoneal (VP) shunts, predominantly in children [3-6], but reports in adults and patients with lumboperitoneal (LP) shunts are extremely rare.

Here, we present a case of an abdominal CSF pseudocyst with an LP shunt, which posed significant diagnostic challenges, along with a literature review.

## Case presentation

**Figure 1:**
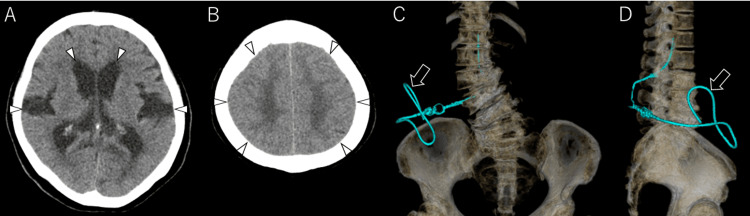
LP shunt for idiopathic normal pressure hydrocephalus (A, B) Preoperative head CT scans reveal ventricular enlargement, widening of the Sylvian fissures, and narrowing of the cortical sulci in the convexity (arrowhead). (C: frontal view, D: right lateral view) Postoperative 3D CT scans demonstrate the distal catheter inserted into the right abdominal cavity following the placement of the LP shunt (arrow).

A 76-year-old woman with a history of unruptured cerebral aneurysm coiling, degenerative lumbar spondylosis, knee osteoarthritis, uterine fibroid surgery in her 30s, sigmoid colon cancer surgery in her 60s, and abdominal wall hernia presented to our outpatient clinic with progressive cognitive decline and gait disturbance. She was diagnosed with idiopathic normal pressure hydrocephalus and underwent LP shunt placement, as she preferred to avoid direct cerebral invasion (Figure 1).

**Figure 2:**
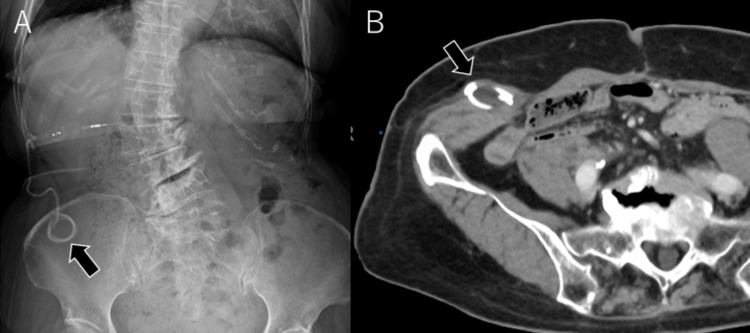
(A) An abdominal X-ray reveals coiling of the peritoneal catheter (arrow). (B) Abdominal CT shows migration of the peritoneal catheter into the subcutaneous tissue (arrow)

Following the procedure, her symptoms improved. However, one year postoperatively, her gait disturbance recurred. Imaging revealed that the peritoneal catheter had migrated into the subcutaneous tissue, necessitating repositioning into the peritoneal cavity (Figure 2).

**Figure 3:**
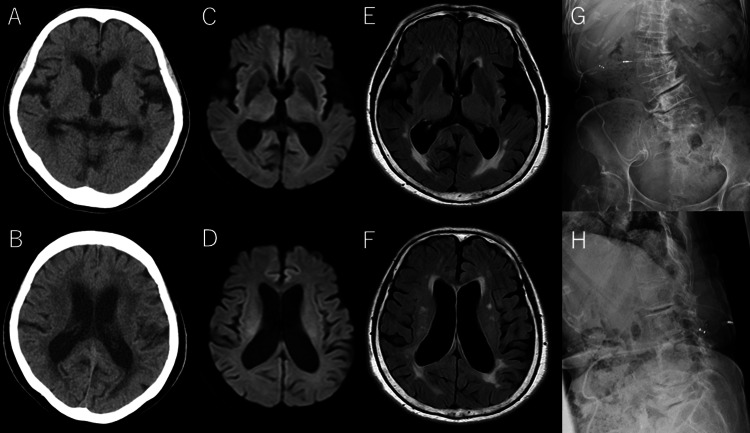
Follow-up imaging studies (A, B) Head CT, (C, D) MRI diffusion-weighted images, and (E, F) MRI FLAIR images show no evidence of new lesions. (G, H) Abdominal X-rays in frontal and lateral views demonstrate no catheter disconnection or migration. FLAIR: fluid-attenuated inversion recovery

Symptoms improved following this revision. However, two years and three months after the initial surgery, her cognitive function and gait disturbances gradually worsened. Brain imaging, including CT and MRI, showed no new lesions, and radiography excluded catheter disconnection or migration (Figure 3).

**Figure 4:**
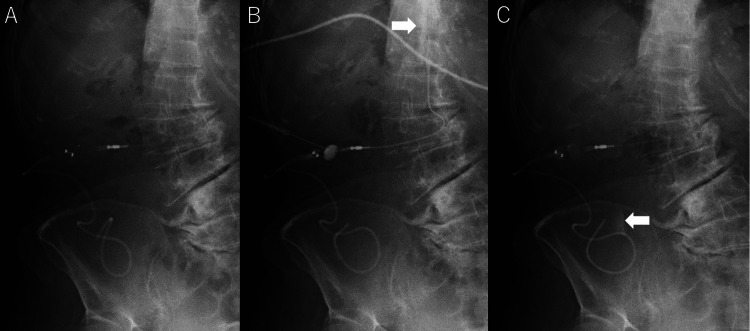
Shuntography (A) The placement of the LP shunt is confirmed. (B) Contrast distribution is shown from the lumbar catheter to the spinal subarachnoid space (arrow). (C) Contrast distribution from the distal catheter to the peritoneal cavity is also shown (arrow).

Shunt function was assessed by puncturing the valve with a 26G needle, revealing normal CSF protein levels and no evidence of infection. Symptoms such as irritability, urinary incontinence, frequent urination, and decreased food intake occurred, prompting shuntography to be performed two years and 11 months after the initial surgery (Figure 4).

**Figure 5:**
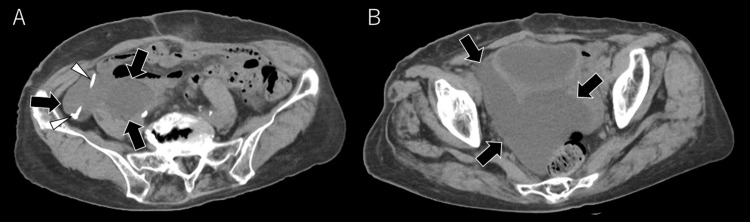
Abdominal CT following shuntography (A, B) Fluid collection is observed from the right paracolic gutter to the vesicorectal pouch (arrows), with the catheter tip within the cyst (arrowheads).

The lumbar catheter showed no obstruction, and the contrast medium flowed into the peritoneal catheter. Contrast medium spreading over the surface of the intestine was not observed (Figure 4C arrow). Abdominal CT revealed a fluid collection extending from the right paracolic gutter to the vesicorectal pouch, with the catheter tip located within the cyst (Figure 5).

This finding confirmed a diagnosis of an abdominal CSF pseudocyst caused by impaired peritoneal absorption due to intra-abdominal adhesions from prior surgeries.

**Figure 6:**
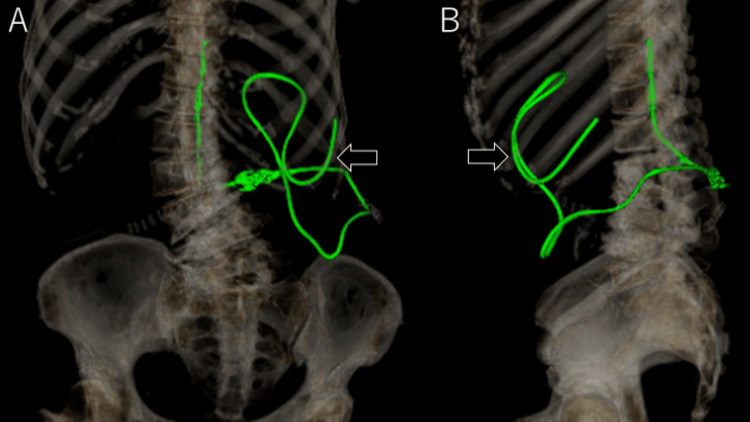
3D CT following LP shunt revision (A: frontal view, B: left lateral view) Post-revision 3D CT scans show the distal catheter inserted into the left abdominal cavity (arrows).

The patient opted against the ventriculoatrial (VA) shunt due to concerns about brain invasiveness. Three years after the initial surgery, the LP shunt system was revised, with the catheter repositioned to the left peritoneal cavity (Figure 6).

Postoperative recovery was uneventful, and symptoms improved. However, due to the risk of recurrence, the patient remains under observation.

## Discussion

Two years after LP shunt surgery, the patient's symptoms worsened, and further investigation revealed an abdominal CSF pseudocyst. Patients with abdominal CSF pseudocysts often present with abdominal symptoms such as abdominal distension or pain, leading them to seek consultation with general internal medicine [7]. The cyst is typically identified by imaging techniques such as abdominal ultrasound and CT scans, with the presence of an abdominal catheter within the cyst being a distinguishing feature. In the present case, the diagnosis was made after suspecting shunt failure, with shuntography and subsequent CT scans confirming the condition. In patients with suspected shunt failure but no obstruction, it is important to consider the possibility of an abdominal CSF pseudocyst. Abdominal CT should be performed to confirm that the abdominal catheter is not positioned within the cyst. Additionally, if no contrast material is observed overlying the intestine on shuntography, the clinician should actively suspect this condition.

The mechanisms underlying the formation of abdominal CSF pseudocysts include the following factors: (1) adhesions caused by abdominal surgery, (2) infection of the CSF and stimulation by protein components in the CSF, and (3) mechanical irritation by the shunt catheter and allergic reactions to the catheter material [7]. These factors likely interact to cause adhesions within the peritoneal cavity, hindering CSF absorption by the peritoneum and ultimately leading to the development of the pseudocyst [7]. Regarding the presence of shunt infection, Mobley et al. reported that if a patient exhibits no fever, normal white blood cell count in blood tests, negative Gram staining of CSF obtained by shunt tap, and normal protein and glucose levels, an infection can be excluded [8]. In this case, no infection was observed in the CSF or cyst fluid, and there were no symptoms suggestive of shunt infection during the clinical course. This case involved a history of multiple abdominal surgeries and an abdominal wall hernia, which resulted in limited mobility of the abdominal catheter in the intraperitoneal space due to adhesions. Consequently, the movement of the abdominal catheter was limited, and mechanical irritation was thought to be the cause of the condition.

In terms of treatment, when intraperitoneal infection is present, temporary external drainage surgery is typically performed, followed by antibiotic therapy before proceeding with shunt reconstruction [3,4,8,9]. In the absence of intraperitoneal infection, options include shunt reconstruction, VA shunt, or ventriculopleural shunt [3,4,8,9]. If a serosal surface without adhesions remains in the peritoneum, a shunt intraperitoneal transition procedure is considered effective [3,6,8,9]. However, in cases with strong adhesions where securing adequate space is challenging, or in recurrent cases, VA shunt or ventriculothoracic shunt should be selected. Although previous reports suggest that LP shunts can be safely placed even in patients with a history of abdominal surgery, preoperative evaluation of peritoneal-intestinal adhesions remains challenging [10]. In this case, although recurrence was a concern, a VA shunt was recommended. However, based on the patient's preference, an LP shunt procedure was performed by inserting the abdominal catheter into the left abdomen, where adhesion was considered minimal.

To the best of our knowledge, only two cases of abdominal CSF pseudocysts associated with LP shunts have been reported, including the present case [11]. Both cases had a history of multiple laparotomies and developed hydrocephalus several years after surgery [11]. Abdominal CT following shuntography revealed the presence of an abdominal CSF pseudocyst [11]. The limited number of reports of abdominal CSF pseudocysts following LP shunt surgery may be attributed to the lower incidence of LP shunts compared to VP shunts, the lower infection rate of LP shunts compared to VP shunts [12], and the greater mobility of the abdominal catheter in LP shunts compared to VP shunts [13].

## Conclusions

We report a case of an abdominal CSF pseudocyst that was difficult to diagnose following an LP shunt. In patients with a history of abdominal surgery, this condition may develop several years after shunt surgery. If shuntography reveals no shunt obstruction and the contrast medium is not detected on the surface of the intestine, the possibility of an abdominal CSF pseudocyst should be suspected, and confirmation with CT is essential. Early detection of this condition is recommended through the proactive use of shuntography and abdominal CT.
